# Patterns and Management of Unprovoked Bull Attack Injuries: A Retrospective Case Series

**DOI:** 10.7759/cureus.33075

**Published:** 2022-12-29

**Authors:** Souvik Maity, Ashok Puranik, Suruthi Baskaran, Metlapalli V Sairam, Prathyusha Kompally

**Affiliations:** 1 General Surgery, All India Institute of Medical Sciences, Jodhpur, IND; 2 Surgery, Government Medical College, Nizamabad, IND

**Keywords:** bull-gore injury, bull-horn injury, emergent general surgery, major trauma, animal attack

## Abstract

Introduction

Animal attacks cause a considerable number of injuries and lead to morbidity and mortality among children and adults. Bull gore injuries following bullfighting and other provoked attacks have been frequently described in literature. Our study describes the pattern of injuries and the unique mechanisms and management of blunt and penetrating trauma associated with unprovoked bull attacks.

Methods

In this retrospective study, we collected the data of 36 patients presenting to our emergency department with a history of bullhorn injury. The data comprised age, sex, location of injury, type and description of the injury, surgical procedure performed if any, requirement of postoperative intensive care unit (ICU) admission, and mortality. The data were then compiled and analyzed with MS Excel.

Results

Among the 36 patients, blunt injuries constituted 58.3% of cases, whereas penetrating injuries were seen in 41.7%. Men were commonly injured with a mean age of 39.1 years. Thorax (36%) and abdomen (33%) were the common sites of injury followed by perineum (17%), head (5%), spine (6%), and extremity (2%). Fall following the impact of bull led to indirect injuries, such as intracranial hemorrhage, parietal bone fracture, cervical spine injuries, and tibial fracture. More than half of the patients (n=19, 52.8%) required some form of surgery under local or general anesthesia. Among the operated patients, seven required postoperative ICU care and two expired.

Conclusion

Animal attack injuries represent a less explored niche of surgical conditions. Management in the emergency department includes prompt resuscitation to achieve hemodynamic stability, thorough wound wash to remove the contaminants, and appropriate imaging, if indicated. Wound exploration is recommended for penetrating injuries and on a case-to-case basis for blunt injuries. The complications of these wounds are due to multiple wound paths, muscle tearing, evisceration of internal organs, and high risk of wound infection.

## Introduction

Bull attacks cause a considerable number of injuries and have become an important public health issue among children and adults. They can be provoked, as seen in bullfighting, or unprovoked, as seen in domestic settings, commonly in the rural areas where livestock are raised. So far injuries reported are mostly from experiences during professional bullfighting [1-3].

The pattern of injuries sustained by provoked and unprovoked attacks are quite different. The mechanism of injuries during bullfighting has been described in the literature as “rag doll” and “spinning top” [4,5]. Bullhorn injuries, though less reported, may cause serious morbidity in the victims and result in the economic burden of healthcare [6,7].

Our study highlights the unique patterns and mechanisms of unprovoked animal attack injuries, which are different from those documented to date. We describe the blunt and penetrating trauma associated with unprovoked bull attacks in a tertiary care center. We also aimed to provide an overview of management and complications of such injuries to add to the existing literature in this less-researched area.

## Materials and methods

In this retrospective study, we collected the data of patients presenting to the emergency department of our hospital with a history of bullhorn injury during the period of January 2018 to July 2021. Clinical data were collected from the electronic patient record system and trauma register. All patients who required admission were included in this study. Injuries caused by direct bullhorn goring as well as indirect impact were included. Road traffic accidents involving collisions between the patient and bull were some examples of indirect impact. Patients with minor injuries who were treated in the emergency department and discharged were excluded. The data collected comprised age, sex, location of injury, type and description of the injury, surgical procedure performed if any, requirement of postoperative intensive care unit (ICU) admission, and mortality. The data was then compiled and analyzed with MS Excel.

## Results

We retrospectively collected the data of patients with the diagnosis of bullhorn injury admitted from January 2018 to July 2021. Among the 36 patients, 35 presented primarily to our emergency department while one of them was referred from another hospital with rib fractures and empyema as a late complication. Majority of injured were males (69.4%). The age of the patients ranged from four to 63 years with a mean age of 39.1 years. The demographic details and clinical course of patients are depicted in Table 1.

**Table 1:** Demographic and clinical history of patients.

Variables		Frequency	Percentage
Gender (n=36)	Male	25	69.4%
	Female	11	30.6%
Age (n=36)	<60 years	29	80.6%
	≥60 years	7	19.4%
Mechanism (n=36)	Blunt	21	58.3%
	Penetrating	15	41.7%
Type (n=15)	Superficial (above fascial planes)	7	46.7%
	Deep (breaching fascial planes)	8	53.3%
Surgery requirement (n=36)	Yes	19	52.8%
	No	17	47.2%
Postoperative ICU admission (n=19)	Yes	7	36.8%
	No	12	63.2%

**Figure 1:**
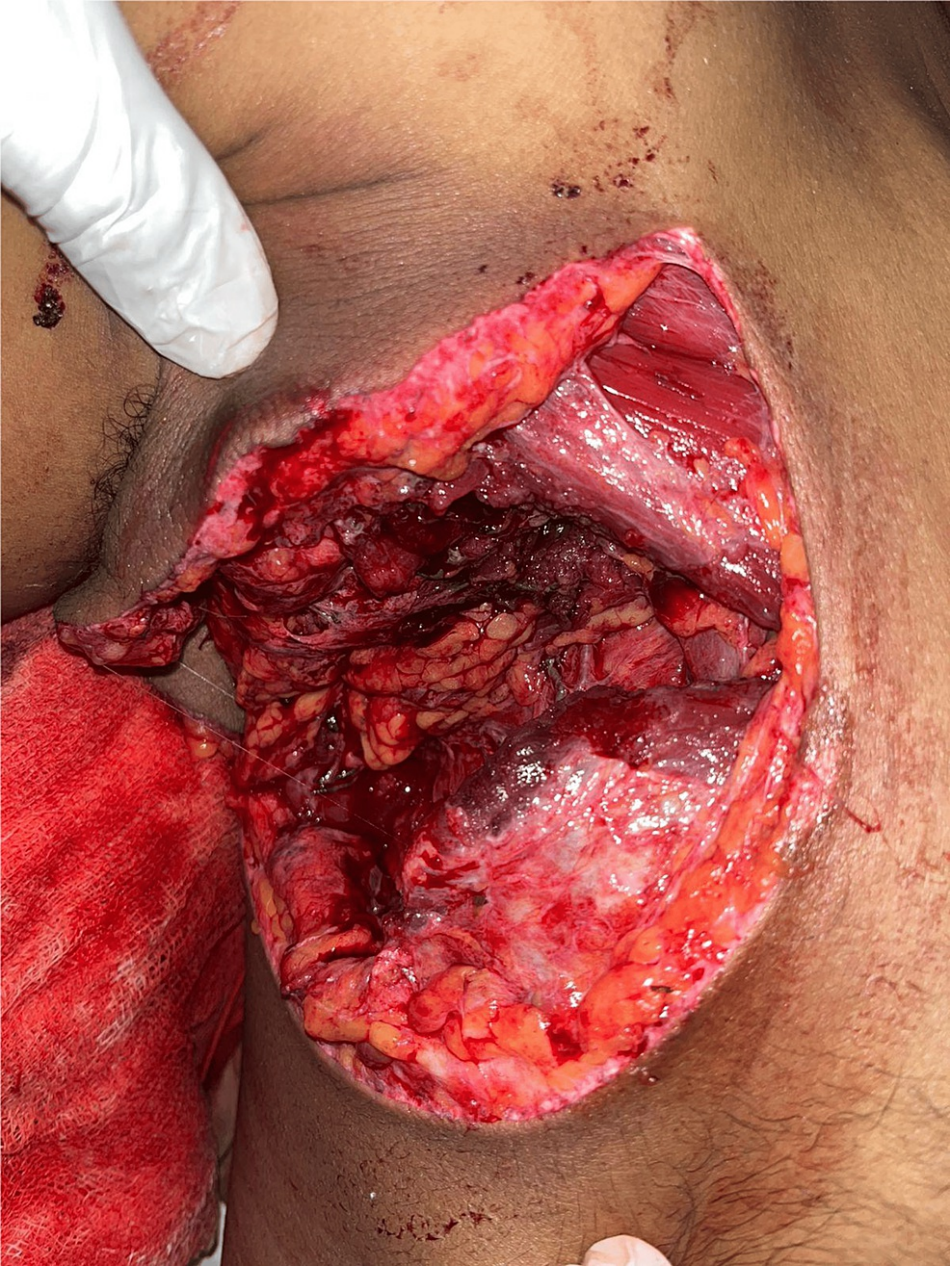
Penetrating injury to right hemithorax with open pneumothorax. Insertion of intercostal drain tube, wound debridement, and closure were performed.

Blunt injuries constituted 58.3% of cases, whereas penetrating injuries were seen in 41.7%. Among penetrating injuries, deep injuries breaching the fascial planes (53.3%) were more common than superficial (46.7%). Figure 1 shows such a deep penetrating injury of thorax with open pneumothorax. Further description of injuries and their frequencies are provided in Table 2.

**Table 2:** Types of injuries encountered in patients and their frequencies. One patient had injuries involving multiple regions like thorax and abdomen and another patient had involvement of cervical spine with necrotizing soft tissue infection of back.

Description of different types of injuries	Frequency
Superficial laceration	7
a. Chest wall	1
b. Abdomen	2
c. Perineum	4
Rib fractures	12
a. Single rib fracture	2
b. Multiple rib fracture	10
Solid viscus injury	7
a. Liver injury	5
b. Splenic injury	1
c. Renal injury	1
Hollow viscus perforation	3
a. Esophageal perforation	1
b. Stomach perforation	1
c. Anal canal perforation	1
Abdominal laceration with bowel evisceration	1
Abdominal laceration with iliac vein injury	1
Scrotal laceration	1
Head injury	2
Bone fractures	1
Spine injury	2
Necrotizing soft tissue infection (NSTI) of back	1

**Figure 2:**
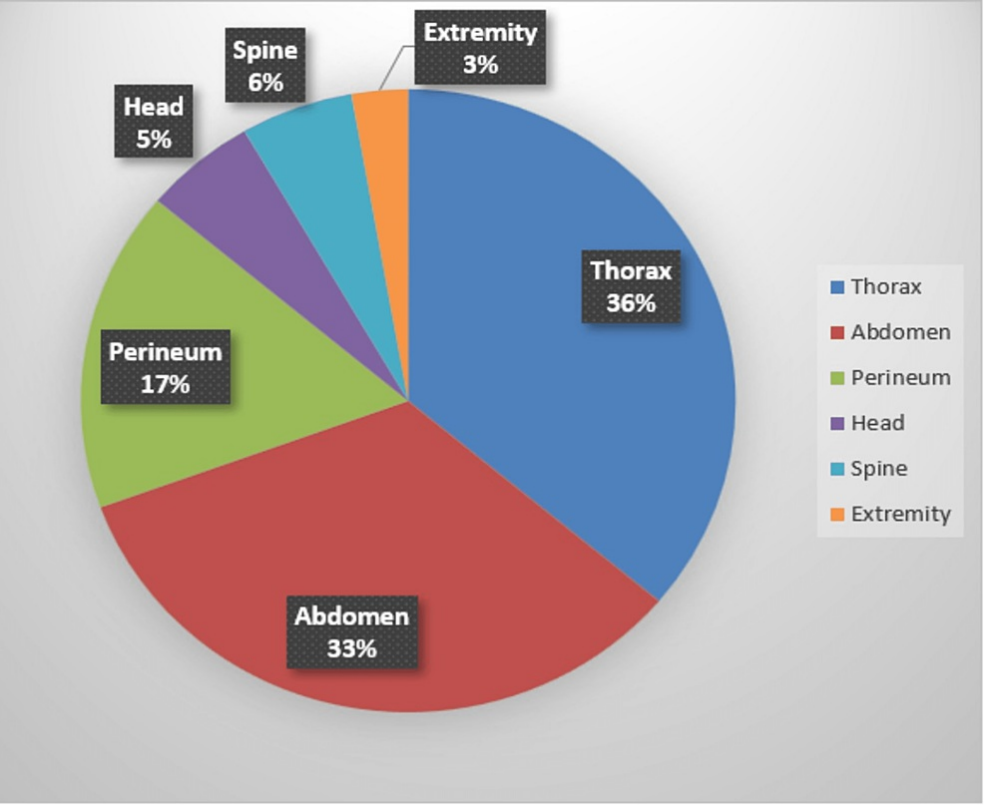
Pie chart showing percentages of location of injury.

Thorax (36%) and abdomen (33%) were the common sites of injury followed by perineum (17%), head (5%), spine (6%), and extremity (2%) (Figure 2). Fall following the impact of bull led to indirect injuries, such as extradural and subdural hemorrhage, parietal bone fracture, cervical spine injuries with or without quadriplegia, and tibial fracture. One patient had injuries involving multiple sites in the form of ninth rib fracture and grade 3 liver injury. We have included that patient under abdominal injury as he was admitted for observation of the liver injury.

Some patients were managed conservatively based on their injury such as rib fractures. However, more than half of the patients (n=19, 52.8%) required some form of surgery under local or general anesthesia. The various procedures performed and their frequency are listed in Table 3. Wound debridement was the most frequent procedure performed. There were several instances where patients required multiple procedures to treat their condition. A patient with perineal wound and anal canal perforation was treated with perineal wound debridement along with diversion colostomy. A patient with quadriplegia due to cervical spine injury presented with necrotizing soft tissue infection (NSTI) of upper back. He underwent a debridement initially, followed by right forequarter amputation in a different setting. One patient with right hepatic duct injury was first taken up for diagnostic laparoscopy and peritoneal wash followed by a postoperative endoscopic retrograde cholangiopancreatography (ERCP) later.

**Table 3:** Different types of procedures performed on patients.

Types of surgical procedure	Frequency
Craniotomy and evacuation of subdural hemorrhage	1
Wound debridement	8
Forequarter amputation	1
Intercostal drain (ICD) insertion	7
Local exploration of open thoracic wound	1
Video-assisted thoracoscopic surgery (VATS) decortication	1
Endoscopic retrograde cholangiopancreatography (ERCP) and stenting	2
Exploratory laparotomy with the below conditions	4
a. Esophageal perforation repair	1
b. Gastric perforation repair	1
c. Liver laceration repair	1
d. Iliac venorrhaphy	1
Diagnostic laparoscopy with peritoneal wash	2
Diversion colostomy	1

Among the operated patients, seven required postoperative ICU care. We had two in-hospital mortality, due to sudden cardiac arrest in ICU in a patient with head injury and septic shock in a postoperative patient with cervical spine injury and necrotizing soft tissue infection of back.

## Discussion

In countries like India, which are more dependent upon livestock, bull and cow horn injuries pose a problem to healthcare. Such injuries are also frequently encountered in countries where bullfighting is practiced as a sport, such as Spain and parts of southern India. The commonly injured people are men of younger working age group followed by middle-aged women who are more often involved in farming in rural India.

The kinematics of provoked animal attacks is typically applicable in the setting of bullfighting. This can also be extrapolated to similar injuries in the domestic setting. However, the mechanisms of unprovoked animal attacks have not been studied in detail. In our study, blunt injuries were more common than penetrating injuries. One of the unique mechanisms was unprovoked bull attacks associated with road traffic accidents, which was observed in three of our patients. All three of them had injuries due to collision with bull while riding a bike. The diagnoses in these patients varied from blunt thoracic injury with oesophageal rupture, penetrating abdominal injury to biliary radicals, and head injury with intracranial hemorrhage. In such cases, the severity of the injuries will depend upon the size, height, and weight of the animal [8,9]. As reported in the literature, animal-vehicle collisions occur late at night on roads with low traffic volumes [10]. Thus, vehicles traveling with high velocity result in high-impact injuries which correlate with the type of injuries these patients had.

Apart from the above mechanism, a raging bull can often drag a person along the ground. This is one of the mechanisms which could not be identified in previous literature. We experienced this with our patient who was attacked by a bull and dragged while working in the field. He presented with a cervical spine injury and later developed a necrotizing soft tissue infection of upper back due to mucormycosis.

**Figure 3:**
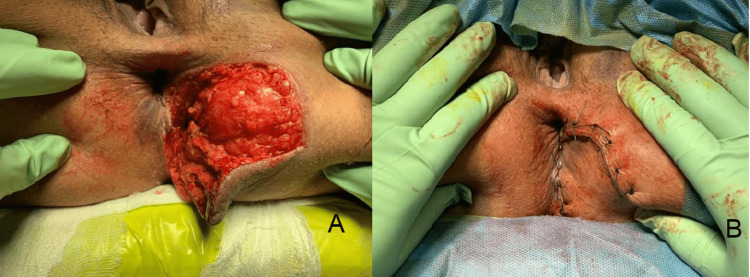
Bull gore injury to perineum with a large laceration (A) and perineal laceration repaired primarily (B). Wound debridement followed by primary closure of the wound was done. The anal sphincter was intact and a diverting colostomy was not performed.

Bull gore injuries also distinguish themselves from other penetrating injuries with respect to muscular tearing, multiple wound paths, distinction between apparent and actual wounds, and foreign body in the wound [11]. Such injuries are also prone to be highly contaminated [3]. Owing to this reason, regardless of the type of wound, local exploration of all the wounds is recommended. The management also becomes challenging due to the complexity of wound and risk of infection. Wound infection due to bull goring is associated with high morbidity [2]. In cases with high contamination, wounds are left open and treated with broad-spectrum antibiotics [12]. Perineal wounds are more susceptible to getting infected due to fecal contamination. Performing a diverting colostomy to prevent fecal soiling of the perineal wound has been commonly advocated [13-15]. However, a clear benefit of colostomy has not been established by randomized studies [16,17]. Early debridement without colostomy in selected cases is also an accepted strategy [18]. In our study, all perineal wounds were managed by early exploration with debridement without a diverting colostomy except in one case that had a full-thickness perforation at the anorectal junction (Figures 3A, 3B). No wound complications were noted.

**Figure 4:**
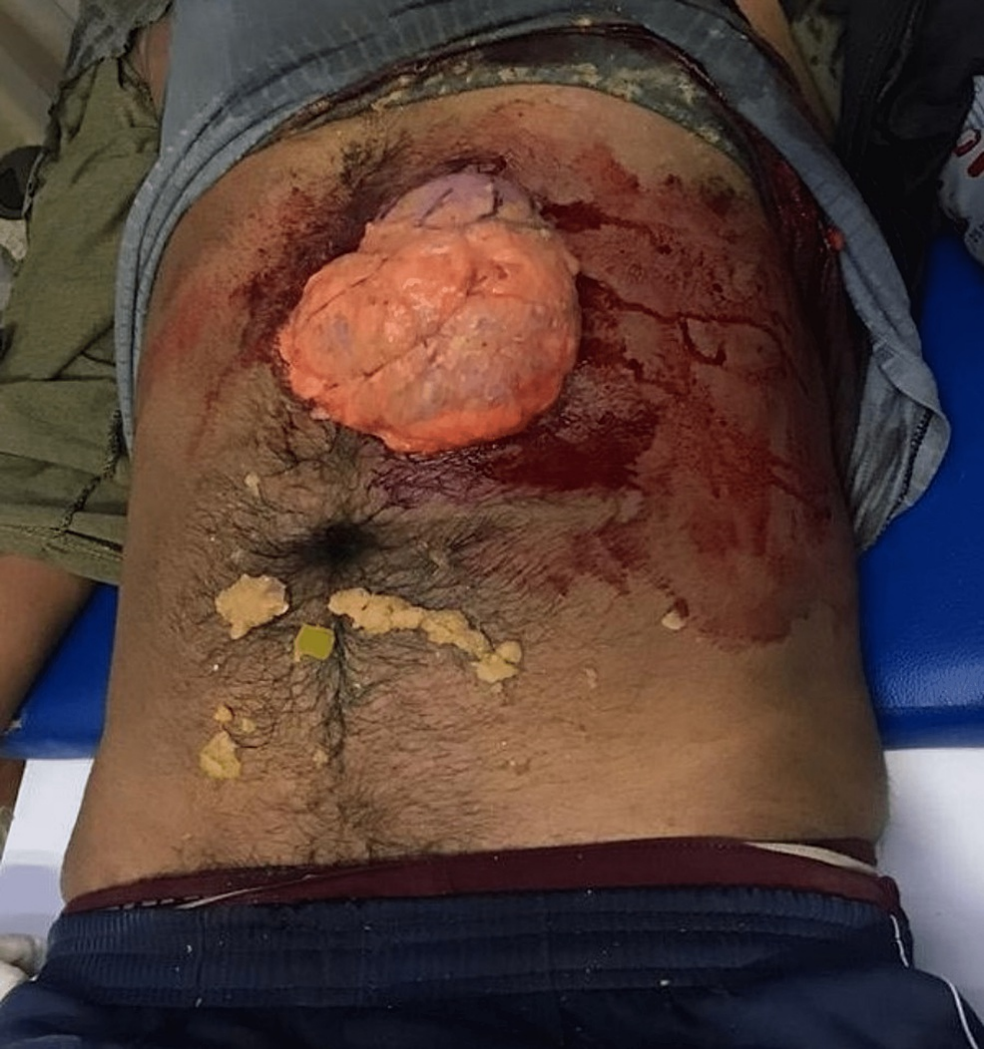
Abdominal wound with evisceration of bowel with no signs of bowel injury.

Abdominal injuries are frequently observed after bull goring accidents. A unique feature of domestic bullhorn injuries is the prolapse of bowel through the abdominal wall without perforation [11]. We had a similar patient with evisceration of bowel from a penetrating wound without any bowel perforation (Figure 4). The abdomen or perineum lies tangentially to the arc of horn movement. So, the injuries more commonly involve superficial structures rather than deep ones.

**Figure 5:**
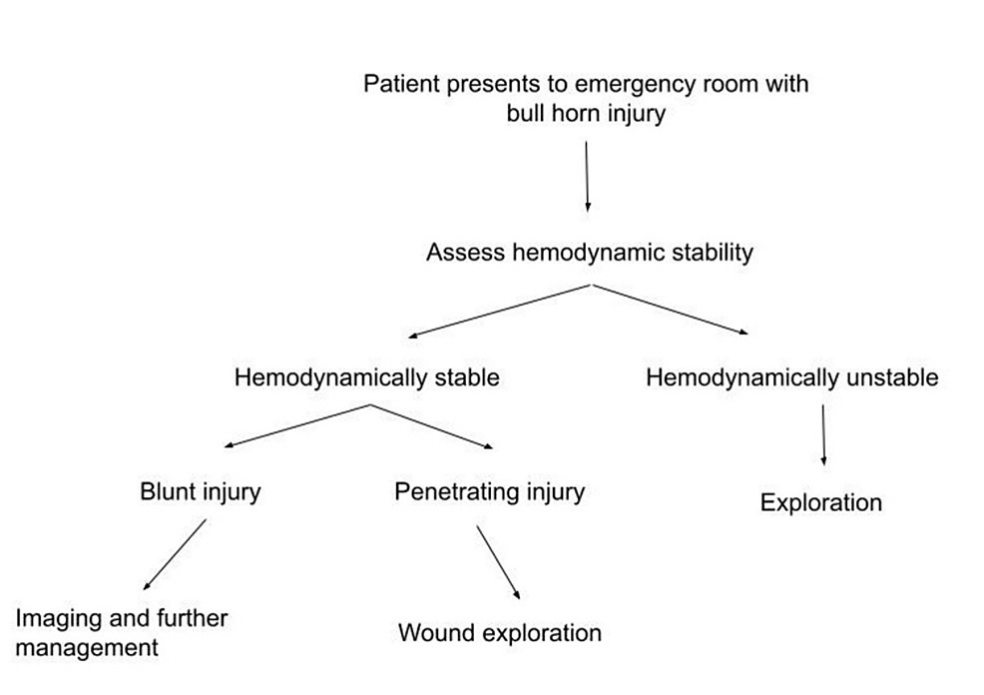
Algorithm for management of bullhorn injuries.

The mortality rates calculated in previous studies range from 0.5% to 1% [2,3]. However, we experienced mortality in 5.6% (n=2) of our patients. The higher mortality could be attributed to smaller sample size. Though injuries due to bull goring could be distracting, the management of such patients should be holistically based on Advanced Trauma Life Support (ATLS) protocols beginning with protection of airway and cervical spine followed by assessment of breathing, circulation, disability, and exposure. A proposed algorithm for management of bullhorn injuries has been depicted in Figure 5.

The limitations of our study include the retrospective nature of the analysis and the small sample size. Long-term prospective studies with a larger sample size would help to overcome this.

## Conclusions

Animal attack injuries represent a less-explored niche of surgical conditions. The attack commonly involves thorax and abdomen, however, sites such as perineum, skull, spine, and extremities can also be affected. The injuries can be blunt or penetrating depending on the type of impact with bull. The complications of these wounds are due to multiple wound paths, muscle tearing, evisceration of internal organs, and high risk of wound infection. Management in the emergency department includes prompt resuscitation to achieve hemodynamic stability, thorough wound wash to remove the contaminants, and appropriate imaging, if indicated. Wound exploration is recommended for penetrating injuries and on a case-to-case basis for blunt injuries. Further studies with a larger sample size and prospective analysis would provide more insight in terms of long-term outcomes of such patients.
